# Behavioral interventions for waste reduction: a systematic review of experimental studies

**DOI:** 10.3389/fpsyg.2025.1561467

**Published:** 2025-06-24

**Authors:** Brent M. Wilson, Magali A. Delmas, Deepak Rajagopal

**Affiliations:** ^1^Institute of the Environment and Sustainability, University of California, Los Angeles, Los Angeles, CA, United States; ^2^Anderson School of Management, University of California, Los Angeles, Los Angeles, CA, United States; ^3^Department of Psychology, University of California, San Diego, San Diego, CA, United States

**Keywords:** systematic review, behavioral interventions, recycling, trash, food waste

## Abstract

**Introduction:**

Wasteful behavior poses major environmental, economic, and social challenges, yet the behavioral science literature on waste reduction remains fragmented.

**Methods:**

This systematic review synthesizes 99 experimental and quasi-experimental studies published between 2017 and 2021 that test behavioral interventions to reduce waste. This period captures a critical phase when global waste management systems faced unprecedented disruptions, including the 2017 launch of China’s National Sword policy, which dramatically reshaped global recycling markets and exposed critical weaknesses in international waste systems. We adopt a broad definition of waste—including both discarded materials (e.g., food, trash, recyclables) and inefficient resource use (e.g., electricity, water, fuel)—to better capture the full range of behaviors where interventions can reduce environmental impact and allow cross-domain comparisons. Our goal is to examine the behavioral interventions used, how interventions are structured, how behavior is measured, and whether they target individuals, households, communities, or broader systems.

**Results:**

We identify six common types of behavioral interventions: education/informational feedback, social norms, economic incentives, cognitive biases and choice architecture, goal setting, and emotional appeals. Interventions targeting electricity and water use were most common, while food and solid waste remain under studied, largely due to measurement challenges. Although most studies used real-world field designs with direct behavioral outcomes, they focused heavily on individual and household behavior.

**Discussion:**

This individual focus risks overlooking the structural and systemic changes needed to achieve broader, sustained reductions in waste. To advance the field, we call for greater use of community-level and system-wide interventions, investment in scalable measurement tools, and stronger collaboration between researchers, governments, and practitioners. Building on this foundation can help create more effective, scalable strategies to reduce waste across behavioral contexts.

## Introduction

Waste is one of the most pressing global challenges of our time. From overflowing landfills and plastic pollution to the invisible waste of energy and water, inefficient resource use has wide-ranging consequences for environmental degradation, economic loss, and social inequality (Kaza et al., 2018; UNEP, 2024; Martuzzi et al., 2010). Waste generation is projected to rise by 70% by 2050 (Kaza et al., 2018), exacerbating pressures on municipal systems, especially in low- and middle-income regions (UN-Habitat, 2010; Wilson et al., 2012). In the U.S., buildings alone account for about 40% of total energy use, underscoring the significant environmental cost of everyday consumption (U.S. Energy Information Administration, 2022).

Despite this urgency, much of the research has focused on technological solutions—such as material recovery or process optimization—while comparatively less attention has been given to the human behaviors that drive waste generation (Islam et al., 2020). A growing body of work recognizes that behavioral change is essential to reducing waste, yet the concept of waste itself remains inconsistently defined. Arkes (1996) emphasized its psychological dimensions, tying waste to feelings of inefficiency, regret, or norm violation. Other scholars describe it as “superfluous,” “careless use,” or “no longer serving a purpose” (Drackner, 2005; Fowles and Nansen, 2020). These definitions suggest that waste includes not only material discards but also the excessive or unnecessary use of resources—such as water, electricity, or fuel—even in the absence of visible residue. This perspective is particularly valuable for behavioral intervention design, as it highlights the everyday decisions and habits that lead to overuse and inefficiency across domains.

Behavioral science provides a robust toolkit to address these behavioral drivers. By targeting factors such as habits, heuristics, social norms, and attentional biases, behavioral interventions aim to shift daily practices in more sustainable directions without requiring structural change (Thaler and Sunstein, 2021; Sunstein, 2014; Dolan et al., 2012). However, the behavioral literature on waste remains fragmented, with wide variation in intervention design, targeted mechanisms, measurement strategies, and levels of analysis (Schultz et al., 1995; Shipley and van Riper, 2022; White et al., 2019).

This systematic review synthesizes 99 experimental and quasi-experimental studies published between 2017 and 2021 that apply behavioral interventions to reduce waste. We selected this period because it captures a critical phase in waste management research, marked by significant global disruptions and regulatory shifts. For example, the 2017 launch of Operation National Sword in China sharply restricted the imports of contaminated recyclable materials, disrupting global recycling markets and highlighting the urgent need for more sustainable, localized waste management solutions. We ended the review in 2021 because studies conducted after this date are likely to reflect the unique, short-term impacts of the COVID-19 pandemic, which significantly altered consumption patterns, waste generation, and recycling systems. These disruptions introduced unprecedented variability that may not be representative of longer-term trends. Unlike prior reviews that focus narrowly on food waste (Casonato et al., 2023; Kim et al., 2019; Srivastava et al., 2023; Zhang et al., 2023), electricity (Abrahamse et al., 2005; Delmas et al., 2013), or recycling (Porter et al., 1995; Varotto and Spagnolli, 2017), we adopt a comprehensive view of waste, encompassing both discarded materials (e.g., food, plastics, trash) and excessive resource use (e.g., electricity, water, fuel). This framing allows us to examine intervention strategies that address both consumption and disposal behaviors, enabling cross-domain comparisons and generalizable insights.

The goals of this review are threefold. First, we categorize the types of behavioral interventions most commonly used to reduce waste, providing a framework to organize this literature. Second, we assess methodological practices, including how behavior is measured (self-report vs. direct observation) and at what level (individual, household, community). Third, we reflect on structural factors that shape this research, such as publication incentives and measurement constraints, which may bias attention toward certain domains or outcomes. Together, these goals aim to support a more integrated understanding of behavioral approaches to waste reduction and to identify opportunities for expanding research across waste domains and levels of analysis.

## Methods

Our systematic review was conducted and reported in accordance with the Preferred Reporting Items for Systematic Reviews and Meta-Analyses (PRISMA 2020) guidelines (Page et al., 2021). Below, we describe the eligibility criteria, search strategy, article selection process, and data extraction procedures used in the review.

### Eligibility criteria

Eligibility criteria for inclusion covered four main areas: study design, behavioral intervention characteristics, outcome measures, and relevant publication attributes.

Specifically, eligible studies had to use an experimental or quasi-experimental design, including randomized controlled trials, field experiments, laboratory experiments, online experiments, or pre-post designs with a comparison group. While field experiments offer the most ecologically valid insights—and we hoped to find many—conducting research in a field setting was not required for inclusion. Studies conducted in laboratory or online environments were eligible as long as they implemented a clear behavioral intervention and employed an appropriate experimental or quasi-experimental design.

To be included, studies had to examine waste-generating or waste-reducing behaviors such as electricity or water conservation, food waste reduction, recycling, composting, fuel use, or general trash generation and contamination. Outcome measures could be observed behavior, self-reported behavior, or behavioral intentions. While intentions are not ideal proxies for actual behavior, they are conceptually and empirically stronger than attitudes (Ajzen, 1991), and their inclusion allowed us to assess the use of direct measures across the literature. This approach is supported by meta-analytic evidence showing a moderate correlation between pro-environmental intentions and behaviors (*r* = 0.508; Helferich et al., 2023). Studies relying solely on attitudinal measures were excluded due to the well-documented attitude–behavior gap (Vermeir and Verbeke, 2006; Claudy et al., 2013).

The included studies could target individuals, households, or communities and had to be published in English between January 1, 2017, and December 31, 2021. We specifically focused on journals within psychology, management, and economics due to their strong theoretical ties to motivation, social norms, cognitive biases, and decision-making. Although this disciplinary focus may have excluded relevant research from fields such as public health, environmental engineering, or education, it allowed for a theory-driven and methodologically coherent synthesis.

### Information sources

Relevant studies were identified by searching the Web of Science Core Collection. The search was limited to peer-reviewed journal articles published between 2017 and 2021, representing the 5 years immediately prior to the start of our coding process. To focus the review on psychology, management, and economics, we used Web of Science category filters to include only journals indexed under Behavioral Sciences, Business, Psychology Multidisciplinary, Management, or Economics. As a result, journals indexed solely under unrelated categories (e.g., engineering, health policy, or clinical science) were excluded. This approach enabled us to capture recent behavioral intervention research that had been published and fully indexed, while keeping the number of articles at a tractable level for deep methodological coding. We supplemented this search with manual reference checks, expert recommendations, and additional articles suggested during the peer review process to ensure comprehensiveness.

### Search strategy

Our search terms were developed to identify behavioral interventions targeting household or community-level waste. We combined general waste-related terms (e.g., “waste,” “trash,” “garbage,” “rubbish”) with domain-specific terms (e.g., “food waste,” “electricity,” “water use,” “recycling,” “fuel,” “composting”) and behavioral terms (e.g., “reusing,” “conserving,” “overconsumption”). To capture relevant research designs, we included keywords such as “behavior change,” “intervention,” “nudge,” “field experiment,” and “randomized control trial.” A complete list of search terms and Boolean operators is provided in the Supplementary material.

### Selection process

The search yielded 586 English-language articles. The lead author conducted an initial screening of all titles and abstracts for relevance. When abstracts were ambiguous, full-text reviews were performed. Two to three trained research assistants independently screened the same articles, classifying each as eligible or ineligible based on the inclusion criteria. Discrepancies were resolved through consensus discussions among the research team. Agreement between the lead author and research assistants was high, with a classification match of 95% and a Cohen’s kappa of 0.83.

**Figure 1:**
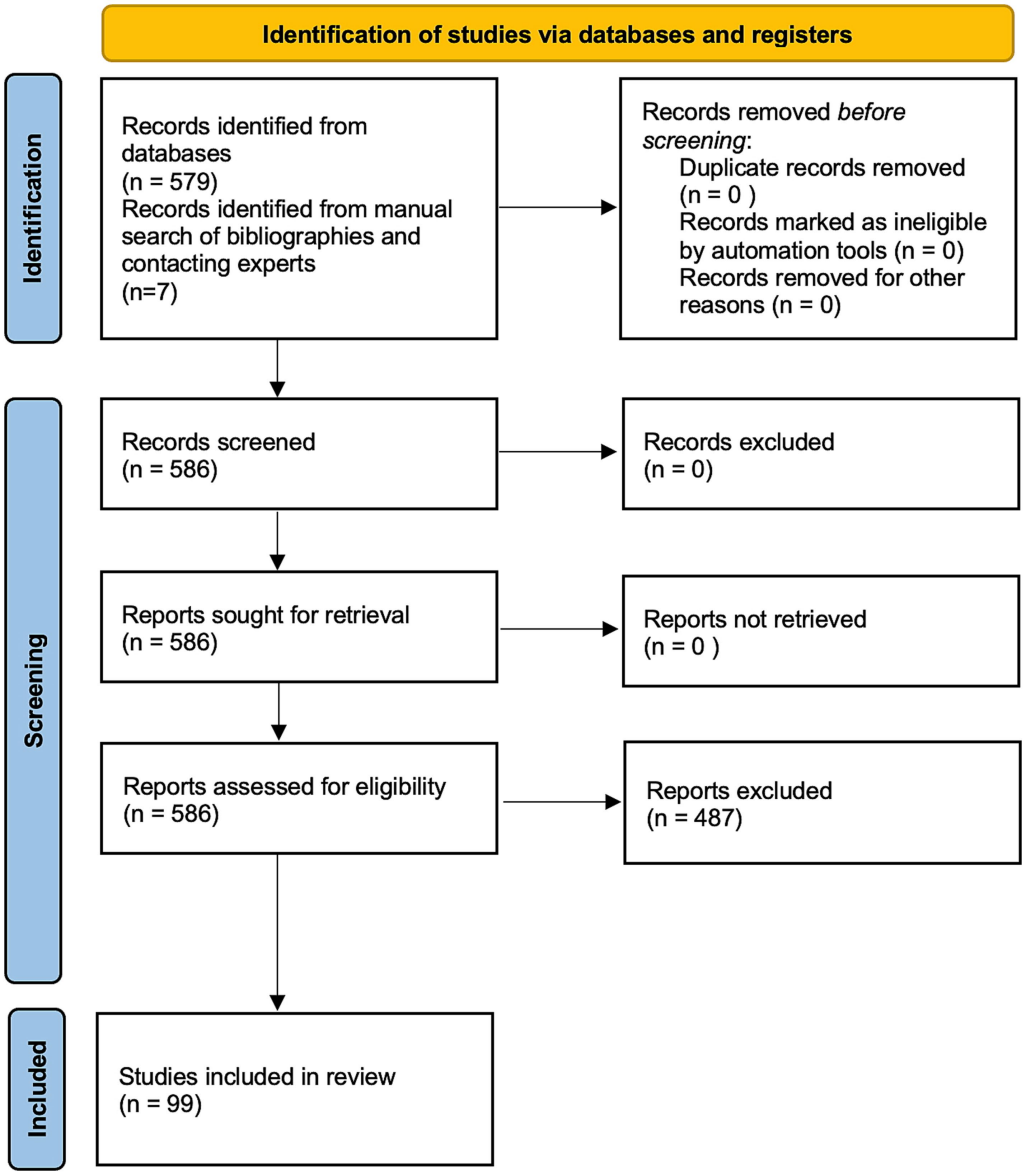
PRISMA 2020 flow diagram of the study selection process. Adapted from Page et al. (2021), BMJ, 372:n71. Licensed under CC BY 4.0.

A total of 99 studies met all eligibility criteria and were retained for full-text coding. Figure 1 presents the PRISMA flow diagram illustrating the screening and selection process.

### Data collection and coding

Each included article was independently coded by trained research assistants using a structured coding protocol. The coding captured variables such as waste domain (e.g., food, energy, water), outcome type (observed behavior, self-report, or intention), unit of analysis (individual, household, community), and experimental design. All initial coding decisions were cross-validated with the lead author, and discrepancies were resolved through discussion and consensus. After initial coding, the dataset was reviewed collaboratively by the author team. Through group discussion and iterative refinement, we developed a classification system for behavioral intervention types based on theoretical grounding and empirical patterns.

### Scope and limitations

This review was designed to provide a theory-driven synthesis of recent experimental research on behavioral waste interventions, with a focus on literature from psychology, management, and economics. While this disciplinary scope offered conceptual and methodological consistency, it may have excluded relevant studies from other fields.

## Results

A spreadsheet of all coded data can be found at https://osf.io/bpyua/.

### Type of waste

**Figure 2:**
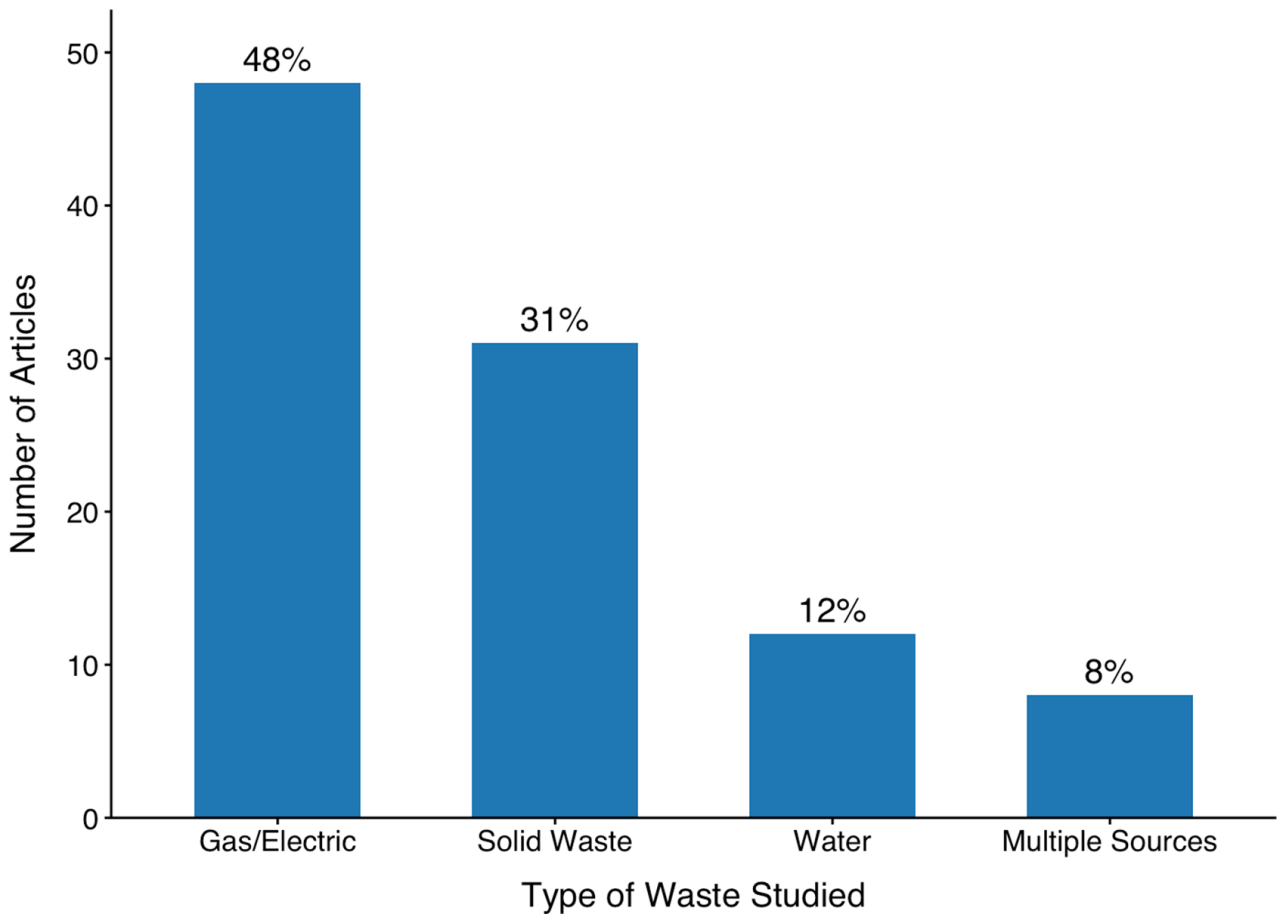
Type of waste studied across the 99 experimental articles included in the review (published 2017–2021). Most studies aimed to reduce gas or electricity use, followed by solid waste and water consumption. A smaller proportion targeted multiple waste types simultaneously.

As shown in Figure 2, the most commonly studied waste is associated with the consumption of gas and electricity (48%), followed by solid waste (31%), water (12%), and a combination of multiple sources (8%). The articles examining *multiple* sources primarily rely on surveys that include various types of waste in a general measure of green behavior. As we discuss in later sections, the high number of articles focused on waste associated with gas and electricity consumption is likely due to the relative ease with which researchers can now get access to real-time data on household consumption from utility companies. The utility company data are accessible and standardized, allowing researchers to study this type of waste more readily than others.

### Dependent variables

**Figure 3:**
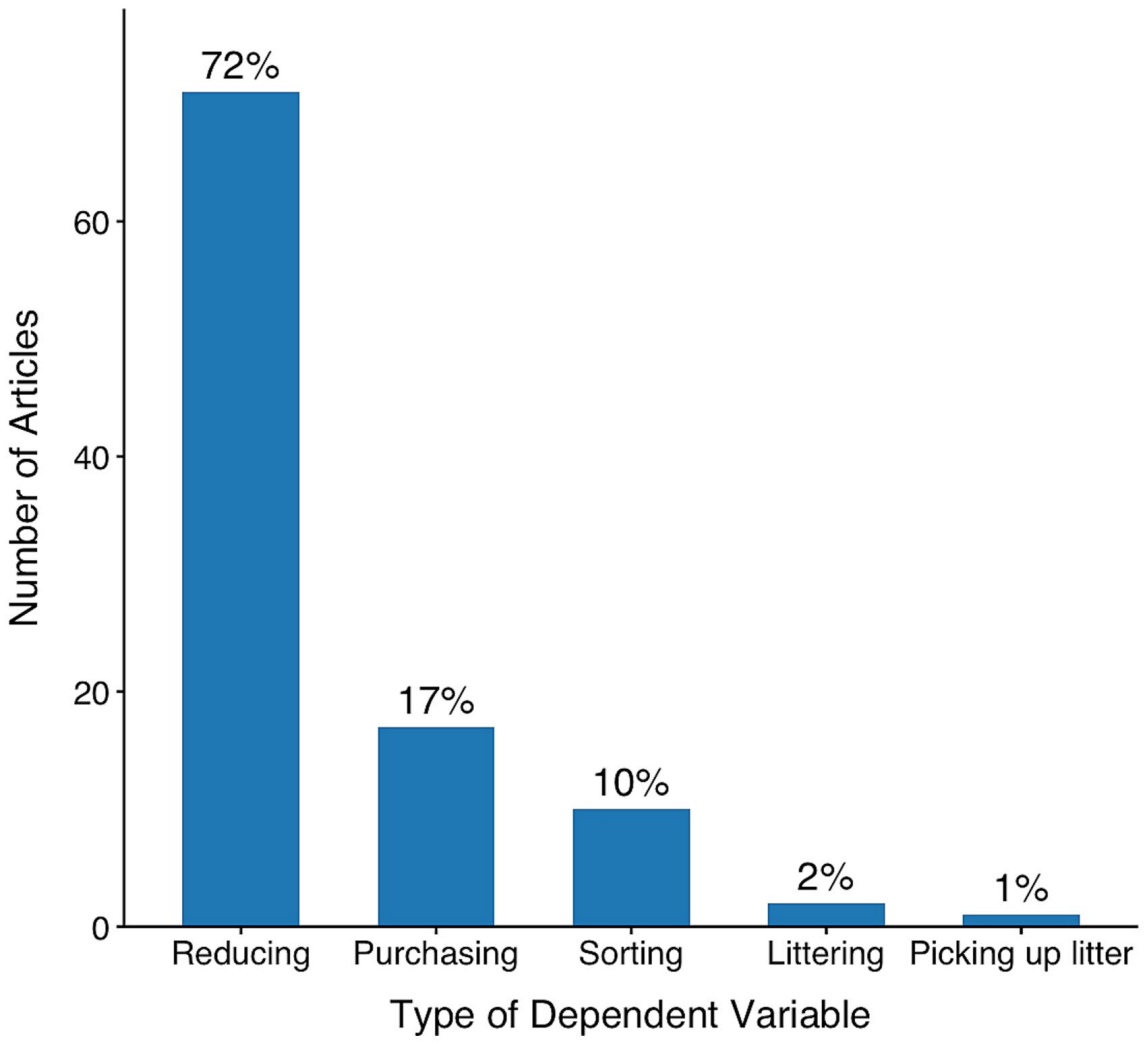
Type of behavior measured in reviewed articles (2017–2021). Most studies evaluated reducing behavior, such as decreasing resource use (e.g., energy, water). Fewer studies focused on purchasing behavior, sorting (e.g., recycling accuracy), littering, or picking up litter.

Most of the articles in our review focus on the reduction of resource consumption or wastage, accounting for 72% of the studies (see Figure 3). Frequent examples include the direct reduction of the amount of electricity or water used by households. Other studies also tend to have the goal of reducing the amount of resources that are used. However, these studies focused on more distal behavior rather than encouraging consumers to directly reduce the amount used. For instance, they target *purchasing* behavior, aiming to decrease wasted resources by promoting the acquisition of energy-efficient appliances (Del Mar Solà et al., 2021) or encouraging the substitution of private vehicle usage with bus trips (Franssens et al., 2021). Additionally, some studies concentrate on *sorting* behavior, seeking to enhance recycling rates (Schwartz et al., 2021) or improving the accuracy of item placement within waste bins (Hayashi et al., 2019). Further aspects include *littering*, encompassing studies that monitor the frequency of illegally disposed garbage bags (Dur and Vollaard, 2019), and pickup behavior, involving studies that observe individuals’ likelihood of *picking up* litter left behind by others (Peck et al., 2021).

### Level of analysis

**Figure 4:**
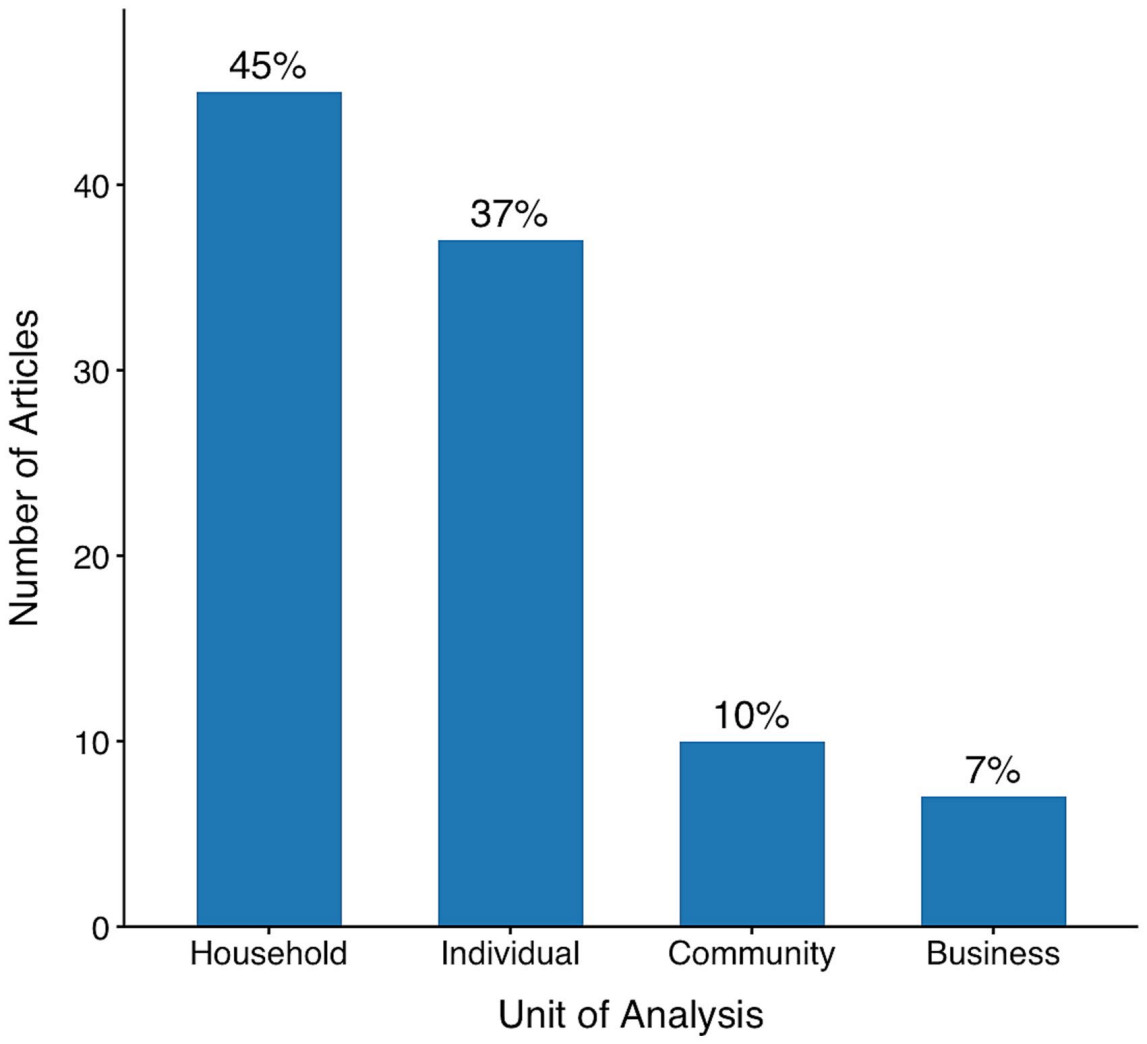
Unit of analysis used in the 99 reviewed studies (2017–2021). Nearly all studies measured behavior at the household or individual level, with relatively few examining behavior at the community or business level.

Most articles focus on behavior at the micro (e.g., individual) or meso (e.g., household, business) levels rather than on the macro (e.g., community) level (see Figure 4). Within the micro level, studies often concentrate on the actions of individuals. For instance, Chaparro et al. (2020) explore the behaviors of bus drivers, and Grewal et al. (2019) examine shoppers’ purchasing patterns. Here, the ‘unit of analysis’ refers to the *individual* being observed, such as a single bus driver or a particular shopper, whose behaviors are measured and analyzed independently of a group or collective entity.

Studies are coded as *household* if the behavior being measured can be attributed to the household as a whole but not to the individual members of that household. Common examples of studies coded as *household* include research examining a household’s electricity (Kniesner and Rustamov, 2018; Chen et al., 2017) or water (Torres and Carlsson, 2018; Brent and Ward, 2019) usage.^1^

Studies coded as *business* examine the combined behavior of multiple employees or customers within a business setting. For example, Del Mar Solà et al. (2021) varied how appliance energy efficiency information was presented across stores and measured the total appliance purchasing behavior at each store. This study is coded as *business* because the unit of analysis captures aggregated customer actions within a commercial environment, not individual choices.

Studies coded as *community* examine the behavior of multiple people who are not clearly confined to a particular business or household. For example, Offiaeli and Yaman (2021) implemented “green lanes” on the London Underground—visual cues indicating where passengers should avoid standing—in an effort to streamline boarding and reduce train delays. This study is coded as *community* because the dependent variable of train delay times is affected by the collective actions of many consumers. As with the distinction between *individual* and *household* studies, the key criterion is whether behavior can be traced to a specific person. If not, and it reflects collective action, the study is coded as *community*.

### Methodology used

Most of the articles rely on actual behavioral measures, are field studies, have a no-intervention control group, and are “true” experiments with random assignment (rather than quasi-experiments without random assignment). Most articles had direct measures of behavior (72%) as opposed to only self-reported behavior or behavioral intentions (Supplementary Table A1). Most articles (85%) also had field studies that were completed in the real world (Supplementary Table A3). Most articles also had appropriate control groups (Supplementary Table A4) and used random assignment (Supplementary Table A5). The average sample size was N = 4,299, and the median sample size was *N* = 336 (Supplementary Figure A1). In addition, most interventions were completed in a day or less (38%), and very few (7%) took longer than a year (Supplementary Table A2). Most studies had participants from North American and European countries (Supplementary Table A6).

### Behavioral interventions

**Figure 5:**
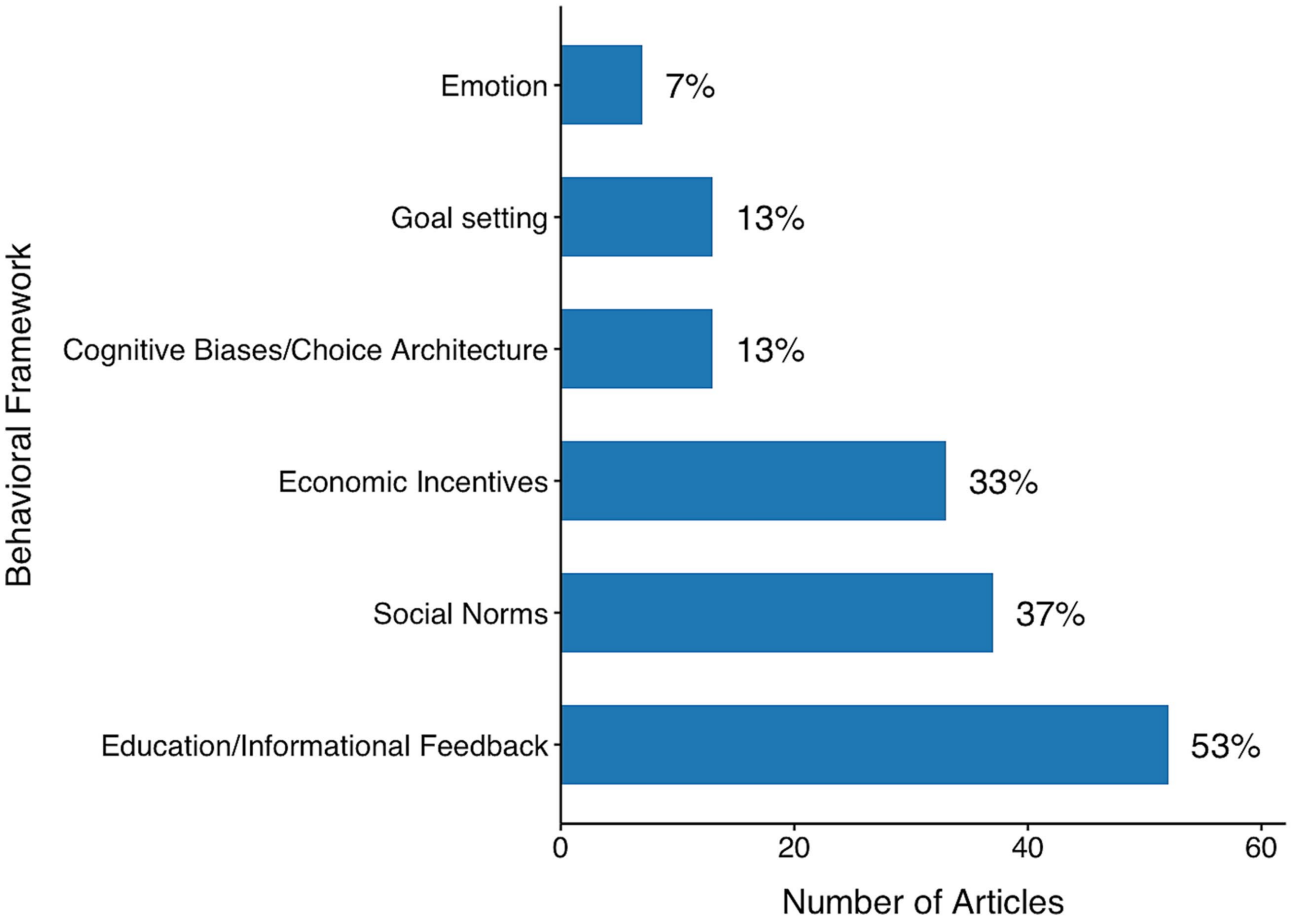
Behavioral frameworks employed in waste-reduction interventions across reviewed studies (2017–2021). The most common strategies involved education or informational feedback, followed by social norms and economic incentives. Less frequently used approaches included cognitive biases/choice architecture, goal setting, and emotion-based appeals.

Using the six-category framework developed during the coding process, we examined how behavioral interventions were distributed across the reviewed studies (Figure 5). These categories consist of education/informational feedback, social norms, economic incentives, cognitive biases/choice architecture, goal setting, and emotion. Each of these categories reflects a distinct theoretical approach to behavior change, which we describe in detail below.

### Education/informational feedback

*Educational or informational feedback* interventions involve providing people with tailored or generalized information about their consumption or waste behaviors to increase awareness and prompt behavior change. This was the most commonly used approach, appearing in 53% of the studies (see Figure 5). However, it was rarely used on its own—77% of studies that included this strategy also employed one or more additional intervention types. This suggests that while information is a useful starting point, it is often insufficient by itself to produce sustained behavior change.

Traditional financial motivators for reducing waste are often minimal and can be undermined by issues such as a lack of awareness or incomplete knowledge (Asensio and Delmas, 2015). Indeed, in wealthy countries, waste is often invisible, and household utilities (like water and electricity) are generally expected to function without fail. When regular blackouts do occur in high-income countries, they are often significant enough to become major news stories, indicating the usual expectation of uninterrupted power supply (Penn, 2022). This lack of visibility and awareness means that people are likely to fail to notice how much they waste or overuse a resource. One potential solution to this problem is providing information about how much is being used so that behavior can be changed (Delmas et al., 2013). Without feedback, people may be unaware that their wasteful behavior deviates from social norms and community standards. Examples of informational feedback include computerized systems for itemizing food waste so that it can then be reduced (Leverenz et al., 2021), in-home displays of electricity usage (Matsukawa, 2018), and how waste bin labels should best convey information about acceptable or unacceptable items (Wu et al., 2018). Leverenz et al. (2021) found that computerized systems that allowed hospitality employees to easily keep track of food waste resulted in a 64% reduction in mass. Relatedly, real-time electricity usage displays reduced consumption by ~1.5% after a month (Matsukawa, 2018). In the recycling domain, the accuracy of sorting waste into the appropriate bin was ~7% higher when signs contained icons or pictures of permitted items (Wu et al., 2018).

However, not all education- or feedback-based interventions can rely on real-time behavioral tracking or direct measurement. In some cases, outcomes must be evaluated through self-reported behavior, which introduces interpretive challenges. An especially striking example is Young et al. (2017), who examined the effects of a public-facing food waste education campaign that included e-newsletters, magazine features, and social media posts encouraging consumers to use leftovers creatively. Participants who had seen these messages reported their food waste behavior in a survey both before and after the campaign. Interestingly, self-reported food waste initially *increased* two weeks after exposure, before decreasing in the subsequent survey conducted several months later. This unusual pattern illustrates the difficulty of using self-report measures for waste behavior. One plausible interpretation is that participants became more aware of their own food waste after the intervention, leading to self-reports shaped by heightened awareness rather than a true behavioral change. However, without objective behavioral data, it remains unclear whether the campaign briefly backfired or whether greater awareness distorted self-reporting. Either way, this study underscores the challenge of interpreting intervention effects when using self-reported data as the outcome measure. That challenge becomes even more pronounced when self-reports are not taken at the time the behavior is performed but instead rely on memory, which is particularly susceptible to reinterpretation. In such cases, it is not necessarily clear that self-reported behavior offers a more accurate measure than behavioral intentions.

Even when studies do measure behavior directly (e.g., by collecting, sorting, and weighing households’ trash), determining the true effectiveness of an intervention can still be tricky. One example is the information-based intervention in Pelt et al. (2020), in which households received a leaflet with tips on how to reduce food waste.^2^ Among the suggestions for reducing food waste while shopping was a recommendation to “check the use-by dates of fresh foods,” which illustrates a broader challenge in evaluating interventions that target individuals or households in isolation. Suppose shoppers become especially attentive to buying foods with the latest use-by dates (and therefore avoid purchasing items that are closer to expiration). In that case, the waste may shift upstream, occurring at the store level rather than within the home. The situation may be even more problematic if the avoided items are still safe to eat but can no longer be sold because they are past the use-by date. This raises a fundamental question about how to define and measure the “success” of an intervention. It also highlights the importance of considering waste at the community level.

### Social norms

The second most common behavioral intervention was *social norms*, appearing in 37% of articles. Social norms are generally considered to be the “rules and standards that are understood by members of a group and that guide and/or constrain social behavior without the force of laws” (Cialdini and Trost, 1998, p. 152). Their power lies in reducing uncertainty about typical or appropriate behaviors in a given context. When people perceive their behavior as deviating from the norm (particularly when others are conserving more), it can trigger a desire to conform, motivated by both informational and affiliative concerns (Cialdini and Trost, 1998). Norm-based interventions are especially effective when the targeted behaviors are public, socially visible, or identity-relevant.

In this review, about half (45%) of the studies that used descriptive norms paired them with injunctive norms, indicating the behavior considered socially desirable (Schultz et al., 2018). Social norms were also paired with education/informational feedback interventions in 57% of the articles where social norms were used. Many of these studies tested variations of the seminal work by Schultz et al. (2007) and Allcott (2011) wherein households are given information about how their electricity or water usage compares to their neighbors’ usage. As might be expected by this type of research design, 62% of the studies motivated by social norms had the household as the unit of analysis. This approach is largely because household utility meter readings offer accessible social comparison data pertinent to this kind of research. These readings are critical as they furnish data for a study’s independent and dependent variables. Barring the use of deceptive practices, which were not evident in any of the studies employing this design, researchers require access to these meter readings. Initial readings are necessary to provide accurate social comparison feedback to participants, while subsequent readings are essential to ascertain the impact of any implemented interventions. All studies (except for one) that used social norm information to reduce household electricity or water usage had meter reading information to verify household consumption. The one exception (Sengupta, 2020) instead relied on self-reported household water consumption, and it was unclear how the researcher obtained social comparison information or if this had also been previously self-reported by households. The reduction in electricity usage achieved by providing informational feedback and social norms tended to be similar to the 2% reduction observed in Allcott (2011). For example, Henry et al. (2019) found a 3% reduction, Sudarshan (2017) a 7% reduction, and Matsukawa (2018) a 2% reduction.

### Cognitive biases and choice architecture

Interventions based on *cognitive biases and choice architecture* aim to guide decisions by altering how options are presented—without restricting choice. These approaches leverage insights from dual-process theories of cognition, particularly the tendency to rely on automatic, intuitive responses (System 1) rather than deliberate reasoning (System 2; Kahneman, 2011). This category represents 13% of the interventions identified in our review (see Figure 5).

Behavior can become more sustainable when the effort required to choose the pro-environmental option is reduced or when intuitive biases that lead to wasteful behavior are corrected. For instance, framing electricity conservation as a potential loss rather than a gain improved outcomes by leveraging loss aversion (Ghesla et al., 2020). Another approach used default settings, such as not automatically placing straws in drinks, which prompted consumers to consciously opt in and reduce plastic waste (Mundt et al., 2020). In the context of food waste, labeling imperfect produce as “ugly” helped counteract aesthetic biases, reminding consumers that such items are still nutritious and safe to eat (Mookerjee et al., 2021). These simple framing strategies corrected biased perceptions and promoted more resource-efficient decisions.

Some interventions addressed friction costs—minor barriers that disproportionately hinder follow-through. Providing a direct, one-click link to download energy feedback reports resulted in a 229% increase in report access (Rosenkranz et al., 2017). Likewise, the introduction of electronic ticketing systems made public transportation more accessible and reduced car usage by 10 min per person per day (Ellison et al., 2017). These examples demonstrate how seemingly trivial design changes can substantially alter behavior.

Other strategies focused on enabling behavior by supplying tools that support waste-reducing actions. One study provided composting kits to households, facilitating food waste diversion and leading to spillover effects on unrelated behaviors such as taking shorter showers or opting for walking and biking. These spillover effects were small to moderate in magnitude, with Cohen’s *d* values ranging from 0.16 to 0.54 (Sintov et al., 2019).

In sum, cognitive biases and choice architecture interventions represent a flexible, often underutilized toolkit for reducing waste. By reshaping decision environments, reducing small barriers, and correcting intuitive misjudgments, these approaches offer scalable, low-cost pathways for encouraging more sustainable everyday behavior.

### Economic incentives

*Economic incentive* interventions aim to promote sustainable behavior by changing its financial calculus—either by increasing the costs of unsustainable actions or by enhancing the benefits of pro-environmental choices. These strategies can include direct financial mechanisms such as subsidies, rebates, penalties, or cost reminders. In our review, this category accounted for 33% of the interventions analyzed (see Figure 5).

According to classical economic theory, individuals make decisions by weighing the expected costs and benefits of different actions. When the perceived benefits outweigh the costs, people are more likely to engage in a behavior. Many of the studies in this category followed this logic by modifying the cost–benefit structure—raising the cost of resource-intensive behaviors (e.g., water or electricity use), offering rewards for reduced consumption, or providing incentives for purchasing sustainable products.

Other studies applied principles from behavioral economics, using subtle cues or reminders to make existing costs more salient or easier to act upon. For instance, text message alerts about high electricity prices during peak hours led to a 14–17% reduction in consumption (Ito et al., 2018). However, not all financial interventions yielded meaningful results. For example, providing information about annual heating costs (Lang et al., 2021) or offering rebate checks for energy-efficient appliances (Schaufele, 2021) showed minimal to no effect, suggesting that awareness alone may be insufficient without perceived immediacy or relevance.

Importantly, the effectiveness of economic incentives may depend on how they interact with intrinsic motivation. Bolderdijk and Steg (2015) caution that while financial incentives can signal that a behavior is socially important, they can also undermine intrinsic motives by framing sustainable choices as externally driven obligations. Once incentives are withdrawn, this shift in motivation can result in reduced long-term engagement.

Future research should explore how incentive design affects not only the initial adoption of sustainable behaviors but also their persistence over time. Understanding when and how incentives complement or conflict with internal motivations will be critical for developing effective and enduring interventions.

### Goal setting

*Goal-setting* interventions prompt individuals to commit to specific, often quantifiable, targets, thereby enhancing behavioral focus and accountability. Thirteen articles (13%) used this strategy, which is effective in part because it translates abstract values into concrete actions.

For example, asking participants to write down specific carbon-reduction actions resulted in a 279% increase in willingness to have their carbon footprint calculated (Parant et al., 2017). Other studies showed that having specific rather than general goals (Bareket-Bojmel et al., 2020) resulted in a 31% reduction in coffee consumption, underscoring how clearly defined goals can effectively shift everyday consumption behavior. Assigning energy savings targets to managers who oversee day-to-day operations, rather than relying on separate volunteers who served as “energy champions,” had a fairly large effect on the likelihood of completing energy checks (Christina et al., 2017; *r* = 0.44). This third example illustrates the importance of signaling that waste reduction is valued by those in one’s immediate environment. If people think that reducing waste is valued only in an abstract sense, they may be unlikely to take concrete actions to reduce it. Encouraging personal commitment can further strengthen goal-setting interventions. For example, hotel guests who signed a pledge to conserve water reduced their usage by 16% per person per night (Joo et al., 2018). Similarly, asking participants to commit to taking the bus for a month led to 56% still occasionally using public transit three months later, compared to 0% before the study (Castel et al., 2019), highlighting the motivational impact of self-commitment.

### Emotion

*Emotional* interventions seek to influence behavior by eliciting affective responses—such as guilt, pride, empathy, or hope—that motivate pro-environmental action. In our review, only a small share of studies (7%) employed emotional appeals, and when they did, these interventions were typically subtle and positively valenced (See Figure 5).

For example, Wang et al. (2017) used cartoon-like “cute” animal imagery on recycling bins, which increased recycling rates by up to 55% compared to standard bins. Similarly, Grewal et al. (2019) found that telling consumers they were “fantastic” for purchasing imperfect produce increased such purchases by 92%, suggesting that even light emotional cues can influence behavior.

Notably, fear-based messaging was almost entirely absent from the studies included in this review. When emotional appeals were used, they tended to focus on uplifting or affirming sentiments rather than fear or guilt. However, even positively framed messages can provoke unintended emotional reactions. Jensen et al. (2020) found that an encouraging message (“Help us find new ways to accelerate the transition to green energy”) increased the emotional cost of saying no, potentially triggering guilt or a sense of obligation.

One of the few studies to use a more negatively valenced emotional appeal was Tiefenbeck et al. (2018), which paired real-time shower feedback with an image of a polar bear stranded on melting ice. The imagery was designed to evoke concern for the climate, though the specific emotional response was not directly measured.

Across the literature, fear appears not as a strategy for change but as a perceived barrier to action. For instance, Ghesla et al. (2020) noted that policymakers may fear backlash or unintended consequences when implementing behavioral interventions. Castel et al. (2019) also observed that individuals may fear trying new behaviors, even if those behaviors are more environmentally friendly, and discussed how interventions could help reduce such apprehension.

Although emotional appeals are often treated with caution, recent scholarship suggests they may be an underused tool for advancing environmental behavior. Brosch (2021) identifies two key pathways through which emotions affect decision-making: shaping appraisals of personal relevance and activating motivational systems. When interventions align with these processes, emotions such as guilt, pride, or hope may be powerful levers for sustainable action. Emotions such as guilt, pride, and hope may thus offer important and underleveraged opportunities to support pro-environmental behavior—particularly when interventions align emotional responses with specific behavioral goals.

Despite their potential, emotional appeals are often treated with caution. Chapman et al. (2017) argue for a more nuanced, science-based approach to emotions in climate communication, emphasizing that emotions shape how people interpret information, assess risks, and make decisions—not just how they are persuaded. Yet in much of the waste behavior literature, the emotional dimension is frequently avoided, perhaps to sidestep controversy. This hesitation may create a blind spot in our understanding of how to drive meaningful change. Waste is not cleaning itself up—and tapping into emotion may be part of the solution.

Together, these findings illustrate the breadth of behavioral strategies currently used to reduce waste and the complexity of designing interventions that translate into meaningful, lasting change. While education and feedback remain the most common entry point, their effectiveness often depends on complementary strategies such as social norms, goal setting, or cognitive nudges. Economic incentives can offer immediate motivation, but may risk crowding out intrinsic engagement, and emotional appeals—though powerful—remain underexplored. Across these categories, interventions frequently rely on intuitive psychological mechanisms, yet their success depends heavily on context, measurement, and design. In the next section, we evaluate how effective these interventions are in practice, and explore the conditions under which they succeed, backfire, or fail to scale.

### Intervention effectiveness

A central aim of this review was to evaluate whether behavioral interventions resulted in measurable reductions in waste-related behaviors. While effectiveness varied by strategy and context, certain combinations of interventions consistently produced positive outcomes. Notably, pairing usage feedback on electricity or water with social norm information emerged as one of the most commonly used and effective approaches. For example, Brent et al. (2020) reported a 38% reduction in water use, Henry et al. (2019) found a 2.9% decrease in electricity use, Jessoe et al. (2021) observed 1.5–2.5% savings during peak hours, and Kažukauskas et al. (2021) recorded a 6.7% reduction in electricity consumption.

However, the success of these interventions often depended on whether participants were financially responsible for their resource use. In a Myers and Souza (2020) study, college students received feedback on electricity use but were not billed individually. In that context, the intervention had no effect—and electricity consumption slightly increased. Although the intervention included education/informational feedback and social norms, the lack of a financial incentive may have undermined its motivational impact.

Overall, harmful effects were rare. Out of 99 studies, only four reported statistically significant negative outcomes. Brent and Ward (2019) found that information about low marginal water costs increased usage. Albalate and Gragera (2020) observed that a parking policy reduced non-resident traffic but increased car ownership among residents. Catlin et al. (2021) showed that labeling bins as “landfill” led to increased mis-sorting of waste. Schwartz et al. (2021) reported that offering participants the option to donate recycling incentives reduced recycling behavior. Some additional studies reported backfire effects for specific subgroups—for example, low-wasting households (Bhanot, 2021)—but did not observe harmful effects at the aggregate level, suggesting limited broader risk.

Whether the high rate of reported success reflects actual intervention effectiveness or is influenced by publication bias remains an open question. Studies with statistically significant findings are more likely to be published than those with null results (Fanelli, 2012). Nonetheless, the absence of widespread harmful effects is somewhat reassuring. If backfire effects were common and substantial, they would likely still appear in the literature despite bias against null findings.

Importantly, publication bias tends to inflate the magnitude of significant effects rather than reverse their direction. False directional findings—known as sign errors—only become likely when statistical power is extremely low (below ~10%; Gelman and Carlin, 2014). None of the reviewed studies appeared to have been published as registered reports, a format in which study protocols are peer-reviewed and accepted in principle before results are known, helping to minimize publication bias and enhance the credibility of effect sizes (Wilson et al., 2020). As such, very large effects (e.g., those exceeding 100%) are likely inflated and unlikely to replicate at the same magnitude. However, the direction of the effect—whether the intervention helped or harmed—is more likely to replicate and provides a more reliable indicator of the underlying relationship.

For this reason, our analysis focuses primarily on the direction of statistically significant results. While individual effect sizes may be unstable, the consistent pattern of beneficial outcomes supports the conclusion that behavioral interventions can play a valuable role in reducing waste-related behaviors.

## General discussion

### Summary of findings

This review synthesized findings from 99 experimental and quasi-experimental studies that tested behavioral interventions aimed at reducing waste-related outcomes. The majority of studies targeted individual or household behaviors, with a strong emphasis on resource consumption—particularly electricity and water use. Fewer studies addressed upstream behaviors such as purchasing or waste sorting. Nearly 80% employed randomized experimental designs, and most were conducted in real-world contexts using direct measures of behavior. This reflects a notable methodological strength in the field, supporting stronger causal inference than earlier correlational studies. While the corporate social responsibility literature has been critiqued for lacking causal research designs (Barnett et al., 2020), our findings suggest that behavioral research on waste has made significant strides in this regard. However, the scope of the literature remains narrow: most studies were conducted in high-income countries, with relatively few examining interventions at the organization or community level.

The focus on studies published between 2017 and 2021 reflects a critical phase in both waste management and behavioral science. This period included major global disruptions, such as the 2017 launch of Operation National Sword, which reshaped global recycling markets, and ended with the onset of the COVID-19 pandemic, which fundamentally altered consumption patterns and waste dynamics worldwide. During this time, researchers increasingly emphasized scalable, data-driven approaches and broader system-level impacts, moving beyond traditional individual behavior change models to include community and organizational interventions. However, this defined five-year window also introduces limitations. While this timeframe allowed for detailed methodological coding and ensured that all studies were fully published and indexed, it does not capture more recent developments in the field. For instance, interventions developed in response to post-pandemic behavioral shifts or advances in choice architecture may have become more prevalent in the years since. This reflects a common challenge in systematic reviews: balancing the need for thorough analysis with the goal of capturing the most current research. Future reviews will need to address these emerging trends to provide a more complete understanding of the ongoing evolution in this field.

Interventions primarily focused on reducing the volume of resources consumed or waste generated. Some targeted related behaviors, such as product purchasing or sorting waste into appropriate bins (e.g., trash vs. recycling), though these were less common. The dominant focus on individual-level action leaves collective or systemic strategies largely underexplored. Encouragingly, most studies measured actual behavior in naturalistic settings rather than relying exclusively on self-reported or hypothetical outcomes. This approach increases the likelihood of producing findings that generalize to real-world implementation. Still, the field remains shaped by the accessibility of certain behaviors and data sources. Much like searching for lost keys under a streetlamp because the light is better, researchers have concentrated on domains that are easiest to measure, leaving many equally important but less visible waste-related behaviors in the dark.

### The measurement of waste

Researchers are well aware that academic journals and reviewers tend to favor articles that feature measurements perceived as reliable, valid, and collected in real-world contexts. This inclination encourages researchers to actively seek and employ such measurements, resulting in their widespread utilization within the recent literature. Unfortunately, not every research question of interest can easily access these types of measurements. Consequently, the scope of questions explored and the research conducted tend to be influenced by the availability of readily accessible measurements.

This challenge is particularly pronounced in waste research, where measurement issues are prominent. For example, we have amassed substantial knowledge regarding household water and electricity usage, but our understanding of food waste disposal remains limited. This discrepancy likely stems partly from stark differences in measurement costs. Water and electricity usage are relatively easy to track due to widespread utility metering, whereas assessing food waste is considerably more complex and costly. It often requires research teams to physically collect, sort, and weigh garbage—and even then, accuracy may be compromised if individuals dispose of food waste down the sink or use other, less visible methods.

Alternatively, households could be tasked with tracking and weighing all food waste, potentially yielding precise measurements. However, these results may not necessarily generalize to future interventions, as they could be heavily influenced by the household’s exceptional effort devoted to monitoring. Furthermore, households willing to engage in such extensive waste monitoring over extended periods may not represent the typical household, further eroding confidence in the findings. This challenge persists even when researchers take precautions to avoid selection bias. For example, in one study, the recruitment materials deliberately avoided any mention of “food waste” to minimize the risk of attracting participants already highly motivated to address the issue (Wharton et al., 2021). Nonetheless, the final sample had baseline food waste levels well below the national average, suggesting that those willing to participate in such a study may still represent a relatively food-efficient and possibly more conscientious subset of the population.

More broadly, rather than grappling with these intricate measurement challenges, recent research often limits its focus to what is simplest and least costly to investigate. This selective approach narrows the scope of inquiry and may miss out on crucial insights due to the constraints imposed by measurement feasibility.

The limited research that does examine solid waste also relies on measures that are not standardized across studies, which poses an additional problem for building a cumulative knowledge base. While virtually all research on electricity consumption measures it in kWh and water in gallons or liters, there is little to no scientific consensus about the most appropriate way to measure solid waste. This gives researchers considerable latitude in how they calculate their dependent variable, which impedes synthesis across studies. It also introduces analytic flexibility, which may encourage researchers to try different data transformations to achieve statistical significance (an increased concern when studies are not preregistered).

A further concern arises when measurements are drawn from a sample of individuals or households rather than the entire community. If study participants differ meaningfully from the average or median community member (as is likely when participation requires significant effort), then the findings may not generalize beyond that sample. This issue is particularly common when individuals opt out of studies or drop out over time, resulting in data that reflect a more motivated and potentially more efficient subset of the population. By contrast, when waste data are collected at the community level, researchers can obtain accurate totals regardless of who participates. For example, if we have aggregate waste measurements for an entire city, we can have greater confidence in those figures than in totals derived from summing individual participant data.

A focus on the individual and household can also place too much emphasis on studying the actions that can most easily be examined at this level, which may not lead to the greatest reductions in community waste. If a trash collection system can readily be implemented to do the sorting, focusing on household and individual sorting behavior may miss the mark on what changes should be taken to solve the problem most effectively. Moreover, the hypothetical scenarios designed by researchers may not be properly aligned with the types of behavior most likely to reduce waste in communities with different infrastructures in place. Getting people to sort trash, recycle, and compost is useless if communities are ill-equipped to separately collect and process these different types of waste. Moreover, achieving scalability for effective behavioral interventions can be challenging because virtually every community employs a distinct waste management and recycling system. Consequently, interventions that prove effective within one community may not yield the same results in a different setting.

While studies examining electricity and waste tend to use similar experimental protocols (such as providing social norms and feedback information to customers) and dependent measures (such as kWh), studies examining solid waste have disparate designs, analyses, and measures. A popular dependent measure for solid waste studies is the percentage of *something*. This ranges from the percentage of households that recycle (Schwartz et al., 2021), the percentage of people picking up trash (Peck et al., 2021), the percentage of cups recycled (Catlin et al., 2021), to the percentage of trash recycled (Mertens and Schultz, 2021). The last one is an especially complex measure for determining the efficacy of an intervention program because it is affected by both the total amount recycled and the total amount of trash generated. For example, with this measure, a household could increase the amount of trash it produces. However, if the proportional increase in the amount recycled is great enough, an intervention that is an abject failure could still be interpreted as a success.

### Incentive structures and the shaping of research practices

A key theme of this review is how incentive structures influence the types of waste behaviors and levels of analysis that researchers study. In academia’s “publish or perish” culture, producing statistically significant results is a key pathway to publication (Grimes et al., 2018; Fanelli, 2010; Bakker et al., 2012). One way to increase the chance of significance (*p* < 0.05) is by boosting statistical power—the probability of detecting a true effect (Cohen, 1992; Button et al., 2013). Power improves most effectively by increasing the sample size, especially the number of participants (Rouder and Haaf, 2018).

For example, collecting one measure per 10 communities yields N = 10. If data are instead collected from 20 households in each, the sample size becomes N = 200—offering far greater power and a higher likelihood of statistical significance. As a result, researchers may favor individual-or household-level studies over community-level ones to increase publishability.

These incentives also make multiple small-scale studies more appealing than a single large-scale intervention, particularly when journals are less likely to publish null results. Running several small studies increases the odds of at least one statistically significant finding. This can discourage investment in larger community-level designs. One potential remedy is the growing use of registered reports, in which journals like *Nature Human Behaviour*, *Cortex*, and *Royal Society Open Science* commit to publication before results are known (Chambers, 2019). This approach treats null results as equally publishable and reduces bias against larger, riskier studies.

When only significant findings are published, interventions may appear universally effective even if they work only for certain groups. The file-drawer effect obscures null results—especially among underrepresented populations—making it harder to identify limits to intervention effectiveness or scale them equitably. While community-level studies are not a complete solution, they offer broader insight than highly localized research and are an important step toward inclusive evaluation.

Finally, our review focused on psychology, management, and economics journals, which may partly explain the strong methodological standards observed. Other disciplines (like environmental science or public health) may emphasize relevance over precision. As Wilson and Wixted (2018) note, fields differ in what they value, and trade-offs between rigor and relevance are often a matter of judgment, not a clear hierarchy.

### Structural barriers and system-level solutions for waste reduction

Behavior is often shaped by the interaction between two fundamental forces: structure—the social, institutional, and material systems that constrain behavior—and agency—the ability of individuals to act freely and independently (Sewell, 1992). While people exercise agency in their daily choices, those choices are frequently limited by broader systemic conditions. For example, even if individuals meticulously sort their waste, their efforts are undermined if the waste management system fails to process it correctly and the items end up in landfills. Similarly, if consumers adjust their electricity use based on peak-time pricing signals, but the energy supplied during those hours is not environmentally cleaner, then the environmental benefit is lost. These examples highlight how individual actions, no matter how well-intentioned, depend on systems-level functionality to be effective.

This interdependence is central to ongoing debates in behavioral science about whether interventions should primarily target individuals (the i-frame) or broader structural and policy contexts (the s-frame; Chater and Loewenstein, 2022). I-frame interventions are typically easier to implement and evaluate but often yield modest, short-term effects. In contrast, s-frame interventions—such as bans on single-use plastic bags (Isbanner et al., 2021)—can produce larger, more sustained impacts because they alter the systems that shape behavior at scale. Our review highlights the importance of restructuring these larger systems to improve behavioral outcomes and enable more comprehensive research and measurement.

Robust data systems are essential for both i-frame and s-frame interventions. However, they are especially critical for s-frame approaches, given their wider scope and potential for unintended consequences. It is vital to track whether system-level interventions actually generate net benefits or introduce new problems. For instance, well-intentioned efforts to shift behavior may fail or backfire if the enabling infrastructure—such as recycling plants or energy grids—is not aligned with the intervention goals.

However, researchers face real constraints in studying these larger systems. In particular, very few studies have measured solid waste directly—likely due to the challenges of obtaining precise, low-cost data. A notable exception is van der Werf et al. (2021), who were able to study solid waste behavior in Ontario, Canada, thanks to a coordinated waste tracking system established by the province (Waste Diversion Ontario, 2015). This effort involved multiple stakeholders, including the Association of Municipalities of Ontario, the City of Toronto, Stewardship Ontario, and the Continuous Improvement Fund. Without such collaboration, the data necessary for rigorous research would not have been available.

These findings align with the multi-level framework proposed by Boulet et al. (2021), which categorizes behavioral influences into the micro level (individual attitudes and habits), meso level (household and community dynamics), and macro level (societal, institutional, and regulatory systems). Although developed in the context of food waste, the framework is useful across a range of environmental behaviors. In our review, most studies focused on the meso level—particularly household behavior—while relatively few addressed the macro-level systems that shape and constrain those behaviors. Future research should integrate these levels more explicitly, recognizing that sustainable behavior change depends not only on individual choices but also on the systems in which those choices occur.

To make this possible, the research community must prioritize the development of measurement infrastructure. System-level interventions are more likely than individual-level ones to produce large-scale effects, which makes rigorous evaluation even more important. Ensuring that such interventions are effective—and not inadvertently harmful—requires data systems capable of tracking outcomes across different domains and levels of analysis.

Collaboration with government agencies, municipalities, and businesses is essential for building this capacity. Many waste-reduction initiatives are already underway, but few are implemented in a way that supports rigorous impact evaluation. One promising strategy is to use random assignment when programs are rolled out gradually. Regions awaiting implementation can serve as control groups, enabling causal inference while ensuring equitable resource distribution. This approach improves research design and avoids structurally disadvantaging specific areas.

Addressing the growing problem of waste will require a much more coordinated and system-focused response than is currently reflected in the literature. While we have learned much about reducing electricity and water use through feedback, social norms, and incentives, these are only part of the solution. In the coming years, more sophisticated infrastructure, stronger data systems, and deeper collaboration will be necessary to expand the scope of behavioral research and support a wider range of community-level and system-level waste reduction strategies.

## Future directions and conclusion

The current body of research on behavioral interventions for waste reduction offers encouraging insights. Many studies demonstrate that relatively low-cost, scalable strategies—such as feedback, social norms, and goal setting—can lead to measurable reductions in resource use. Methodological rigor has also improved, with a growing number of field-based studies using direct behavioral outcomes.

Still, important gaps remain. Future research should explore how structural systems—such as waste-tracking infrastructure or regulatory environments—influence both the behaviors studied and the interventions designed. Comparative studies across regions with different data systems could illuminate the enabling conditions for high-quality waste research, particularly for domains like solid waste that remain underrepresented.

A second priority is to investigate how institutional reluctance to measure or share performance data affects the evaluation and scaling of interventions. While data gaps and coordination challenges have historically limited transparency, recent advances in information technology and artificial intelligence offer promising pathways to reduce the cost of data collection, integration, and analysis. These tools may help unlock greater access to performance metrics and enable more timely, comparable, and actionable insights across organizations.

Third, future studies should move beyond individual-level (i-frame) interventions to test systemic (s-frame) strategies—such as bans, pricing reforms, or infrastructure changes—alone or in combination with behavioral nudges. For instance, randomized evaluations of municipal pay-as-you-throw pricing schemes could help assess how dynamic pricing and real-time feedback affect household waste generation. Another exciting direction involves testing smart-bin infrastructure equipped with AI-driven contamination alerts to reduce improper recycling and increase compliance. Researchers should also take advantage of opportunities to evaluate real-world programs using randomized rollout designs, which are especially well-suited to examining how large-scale policy or infrastructure shifts shape behavior across diverse settings and populations. These directions can help build a more comprehensive, scalable, and system-aware science of waste behavior—one that meets the scale of the problem we aim to solve.

## Data Availability

The datasets presented in this study can be found in online repositories. These data can be found here: https://osf.io/bpyua/.

## Footnotes

^1^If these studies had given each household member a separate meter to record individual utility use instead of measuring household-level utility use, the study would have been coded as *individual* rather than *household*. *Individual*, therefore, does not necessarily mean that the behavior occurs outside of the home. Rather, the distinction refers to the most granular level at which data were collected.

^2^Although the authors treated the information-based condition as a control group, the descriptive data in Table 3 of Pelt et al. (2020) show that it had the lowest average levels of food waste per person at both follow-up points.
