# Supplementary material for: Behavioral interventions for waste reduction: a systematic review of experimental studies
Source: Front Psychol. 2025 Jun 24;16:1561467. doi: 10.3389/fpsyg.2025.1561467 (PMC12236104; doi:10.3389/fpsyg.2025.1561467)
Supplement: Supplementary file 1 [file Data_Sheet_1.PDF]

SUPPLEMENTARY MATERIAL

Appendix 1. Detailed Eligibility Criteria

We applied the following inclusion and exclusion criteria to determine which studies were eligible for inclusion in the review. These criteria guided the article screening, data extraction, and analysis process.

Inclusion Criteria

Articles were included if they met all of the following conditions:

- **Study Design**
  The study used an experimental or quasi-experimental design. This included:
  - Random assignment to experimental and control groups, or
  - A quasi-experimental or natural experiment with behavioral measurements taken both before and after the intervention.
    Studies that included only post-intervention measurement and lacked a control or comparison condition were excluded.
- **Intervention Focus**
  The intervention was designed to reduce waste-related behavior at the individual, household, or community level. Waste-related behavior was broadly defined to include:
  - Electricity, water, or fuel usage
  - Food waste or trash generation
  - Green purchasing behavior
  - Recycling accuracy or contamination rates
- **Outcome Measures**
  The study included at least one behavioral or behavioral intention outcome:
  - Behavioral outcomes included both objectively measured (e.g., utility usage data, trash weights) and self-reported behaviors.
  - Behavioral intentions, such as willingness to pay or stated likelihood of engaging in a future behavior, were included. Studies that assessed only attitudes, beliefs, or perceptions were excluded.

- **Publication Criteria**
  - Articles were published in peer-reviewed journals
  - Publication years: 2017 to 2021
  - Language: English
  - Journals must be indexed in Web of Science and classified under psychology, management, economics, or closely related disciplines. These categories were selected to align with the behavioral science frameworks guiding the review. In accordance with the review protocol, articles identified through manual searches or expert recommendations (e.g., during peer review) could also be included, provided they met all other inclusion criteria. A full list of journals included is provided in Appendix 3.

Exclusion Criteria

Articles were excluded if they:

- Used purely correlational or cross-sectional designs
- Lacked a behavioral or intention-based outcome measure
- Measured only attitudes, perceptions, or beliefs
- Lacked a control group or a pre-post comparison
- Focused only on policy, education, or infrastructure without a behavioral component
- Were non-empirical (e.g., reviews, theory articles, protocols, or commentaries)
- Were published in journals outside the core disciplinary fields of psychology, management, or economics

Appendix 2. Word search

AB=(waste OR garbage OR rubbish OR trash OR food waste OR energy efficien* OR “water us*” OR ”public transport*" OR "vehicle miles travel*" OR "motor car use" OR recycl* OR reus* OR conserv* OR compost* OR overconsumption)

AND AB=(intervention OR field experiment OR randomized control trial OR behavior chang* OR nudg* OR “choice architecture”)

Appendix 3. List of journals included in the analysis

American Economic Journal-Economic Policy; Applied Economics Letters; Behavior and *Social Issues; Behavioral Sciences; Current Psychology; Economic Inquiry; Economics Letters; Ecopsychology; Energy Economic; Energy Journal, Energy Policy; Energy Research & Social Science; Environment* and *Behavior; Environmental & Resource Economics; Frontiers* in *Psychology; Global Business Review; Human Resource Management Journal; Industrial Marketing Management; International Journal* of *Consumer Studies; Journal* of *Benefit-Cost Analysis; Journal* of *Consumer Marketing; Journal* of *Consumer Policy; Journal* of *Consumer Psychology; Journal* of *Economic Behavior & Organization; Journal* of *Economic Psychology; Journal* of *Environmental Economics And Management; Journal* of *Environmental Psychology; Journal* of *Marketing; Journal* of *Marketing Management; Journal* of *Organizational Behavior Management; Journal* of *Policy Analysis And Management; Journal Of Public Policy & Marketing; Journal* of *The Association* of *Environmental* and *Resource Economists; Management Science; Omega-International Journal* of *Management Science; Organization & Environment; Organizational Behavior* and *Human Decision Processes; Proceedings of the National Academy of Sciences; Psychology & Marketing; Regional Science* and *Urban Economics; Research in Transportation Economics; Resource* and *Energy Economics; Resources, Conservation & Recycling; Scandinavian Journal* of *Economics; Social Marketing Quarterly; Sustainability; Tourism Management; Transport Policy; Transportation Journal; Transportation Research Part A-Policy* and *Practice*

Appendix 4. Coding Criteria

We applied the following coding criteria to extract and classify study-level data for all eligible articles included in the systematic review. These criteria guided the structured assessment of study context, intervention design, behavioral outcomes, and experimental methodology. Each criterion corresponds to a column in the coding dataset at https://osf.io/bpyua/.

Article (Column A)

Author name(s) and year

Type of Experiment (Column B)

Describes the setting and whether participants were aware of being in a study:

- *In-lab*: Conducted in a laboratory setting; participants knew they were in a study.
- *Online*: Conducted via an online platform (e.g., MTurk); participants knew they were in a study.
- *Real-world aware*: Conducted in a natural setting; participants were aware of their participation.
- *Real-world unaware*: Conducted in a natural setting without participants' awareness of being studied.

Unit of Analysis (Column C)

Specifies the level at which behavior was measured and analyzed:

- *Individual*: Behavior measured at the single-person level.
- *Household*: Outcome measured at the collective household level.
- *Business*: Aggregated behavior of employees or customers within a business.
- *Community*: Behavior measured across public or shared spaces, involving multiple unrelated individuals.

True Experiment vs. Quasi-experiment (Column D)

Indicates the use of random assignment:

- *Experiment*: Participants (or units) were randomly assigned to conditions.
- *Quasi-experiment*: No random assignment; includes natural experiments or before–after comparisons.

Independent Variable (Column E)

Describes the specific variable manipulated by the study (e.g., feedback message, default change, price discount). This field is recorded as a free-text entry.

Type of Waste (Column F)

Specifies the type of environmental waste targeted by the intervention:

- *Gas/Electric*: Energy use such as electricity, gas, or heating.
- *Solid Waste*: Trash, food waste, or recyclable materials.
- *Water*: Water consumption or conservation.
- *Multiple Sources*: Interventions targeting more than one waste type (e.g., combined energy and water use).

Dependent Variable (Column G)

Specifies the outcome being measured (e.g., kWh of electricity used, gallons of water, pounds of waste). This is recorded as a free-text field.

Type of Dependent Behavior (Column H)

Describes the nature of the target behavior:

- *Reducing*: Consuming fewer resources.
- *Purchasing*: Anything having to do with choosing to buy a product or service.
- *Sorting*: Anything having to do with disposing of waste in one manner rather than another.
- *Littering*: Leaving waste in undesignated areas.
- *Picking up litter*: Collecting others' discarded waste.

Actual Behavior or Behavioral Intention (Column I)

Captures the level of behavioral measurement:

- *Measured Behavior*: Directly observed or verified (e.g., utility data).
- *Self-reported Behavior*: Based on participants' self-assessment.
- *Behavioral Intention*: Hypothetical responses to imagined scenarios (e.g., willingness to pay, planned behavior).

Control Group (Column J)

Indicates the type of comparison used:

- *No Intervention*: Compared to a group receiving no intervention.

- *Other Intervention*: Compared to a different intervention.
- *Baseline*: Compared to the same group's pre-intervention performance.

Timeline (Column K)

Specifies how long the intervention itself lasted for the unit of analysis:

- *Day*: 1 day or less.
- *Week*: More than 1 day but 1 week or less.
- *Month*: More than 1 week but 1 month or less.
- *Year*: More than 1 month but 1 year or less.
- *Year+*: More than 1 year.

Effectiveness (Column L)

Indicates the direction and magnitude of the intervention effect:

- *Beneficial Effect*: Significant improvement in environmental behavior.
- *Harmful Effect*: Significant increase in waste.
- *Beneficial (Negligible)*: Positive trend, not statistically significant or very small.
- *Harmful (Negligible)*: Negative trend, not statistically significant or very small.
- *No Effect*: No measurable or reported change.

Nudge or Real Incentive (Column M)

Identifies the mechanism of behavioral influence:

- *Nudge*: Alters cognitive environment without changing material incentives.
- *Real Incentive*: Provides tangible costs or benefits (e.g., rebates, price changes).

Country (Column N)

The country in which the intervention took place.

Continent (Column O)

The corresponding continent of the study's location.

N (Column P)

The reported sample size, including any units (e.g., "300 households").

N.no label (Column Q)

The numeric value of the sample size (e.g., 300).

Intervention Strategy Coding (Columns R–W)

Each of the following columns represents a specific type of behavioral strategy used in the intervention. For each study, mark an "X" in the appropriate column(s) if the strategy applies. Multiple strategies may be marked per intervention.

- **Social Norms (Column R):**
  Mark if the intervention uses information about others' behaviors, approval, or social influence to shape behavior through perceived social expectations (e.g., "your neighbors recycle more than you," public commitments, peer comparisons, or observing others model the behavior).
- **Education / Informational Feedback (Column S):**
  Mark if the intervention provides factual, contextual, or personalized information (e.g., utility usage, waste statistics) intended to raise awareness and promote behavior change.
- **Cognitive Biases / Choice Architecture (Column T):**
  Mark if the intervention modifies the decision-making environment to influence behavior (e.g., setting defaults, framing choices, simplifying options).
- **Economic Incentives (Column U):**
  Mark if the intervention involves monetary rewards, penalties, discounts, or pricing mechanisms designed to motivate behavioral change.
- **Emotion (Column V):**
  Mark if the intervention appeals to emotions (e.g., pride, guilt, empathy) as a motivator for pro-environmental behavior.
- **Goal Setting (Column W):**
  Mark if the intervention encourages participants to commit to specific targets, plans, or pledges related to behavior change.

Type of Social Norm (Column X)

Complete this column only if Column R (Social Norms) is marked with an "X." Select the specific type of social norm used in the intervention:

- *Descriptive:* Communicates what others are doing to influence behavior (e.g., "Most people in your area recycle").
- *Injunctive:* Emphasizes what others approve or disapprove of, highlighting social values or norms (e.g., "Recycling is the right thing to do").
- *Descriptive + Injunctive:* Combines what others do and what they approve of (e.g., "Most people recycle and think it's important").
- *Social influence:* Relies on interpersonal pressure, group dynamics, or public visibility (e.g., peer comparisons, social accountability).
- *Modeling behavior:* Demonstrates the desired behavior through example, allowing others to imitate it (e.g., seeing someone properly dispose of waste).

Source of Inclusion (Column Y)

Indicates how the article was identified for inclusion in the review:

- *1:* Article was retrieved from the original Web of Science search and included through the systematic screening process.
- *0:* Article was added later through manual searches or expert recommendations (e.g., during peer review), but still met all inclusion criteria.

Bibliographic Information (Columns Z and Beyond)

Columns Z and onward include bibliographic and indexing details automatically pulled from the Web of Science database for each article. These fields were not manually coded but were retained for reference and filtering.

Appendix 5. List of articles included in the analysis

Article	Education/ Informational feedback	Social norms	Economic incentives	Cognitive biases/ Choice architecture	Goal setting	Emotion	# of Intervention Frameworks
Chwialkowska et al. (2021)	●						1
Clayton et al. (2021)	●						1
Hoffmann & Thommes (2020)	●						1
Kim et al. (2020)	●						1
Kniesner & Rustamov (2018)	●						1
Leverenz et al. (2021)	●						1
Linder et al. (2018)	●						1
Marshall et al. (2019)	●						1
Samotyja et al. (2021)	●						1
Wharton et al. (2021)	●						1
Wu et al. (2018)	●						1
Young et al. (2017)	●						1
Bhanot (2021)		●					1
Czajkowski et al. (2019)		●					1
Franssens et al. (2021)		●					1
Haghighatjoo et al. (2020)		●					1
Kazukauskas et al. (2021)		●					1
Lede et al. (2019)		●					1
Loschelder et al. (2019)		●					1
Mertens & Schultz (2021)		●					1

Article	Education/ Informational feedback	Social norms	Economic incentives	Cognitive biases/ Choice architecture	Goal setting	Emotion	# of Intervention Frameworks
Offiaeli & Yaman (2021)		●					1
Sengupta (2020)		●					1
Torres & Carlsson (2018)		●					1
Wong-Parodi et al. (2019)		●					1
Chaparro et al. (2020)			●				1
Hoffmann & Thommes (2020)			●				1
Isbanner et al. (2021)			●				1
Ito et al. (2018)			●				1
Kaiser et al. (2020)			●				1
Schaufele (2021)			●				1
Schwartz et al. (2021)			●				1
Wang et al. (2021)			●				1
Friman et al. (2019)				●			1
Ghesla et al. (2020)				●			1
Hoffmann & Thommes (2020)				●			1
Kamleitner et al. (2019)				●			1
Mookerjee et al. (2021)				●			1
Mundt et al. (2020)				●			1
Peck et al. (2021)				●			1
Agarwal et al. (2017)					●		1
Bareket-Bojmel et al. (2020)					●		1
Bergquist et al. (2017)					●		1
Christina et al. (2017)					●		1

Article	Education/ Informational feedback	Social norms	Economic incentives	Cognitive biases/ Choice architecture	Goal setting	Emotion	# of Intervention Frameworks
Clayton & Nesnidol (2017)					●		1
Parant et al. (2017)					●		1
Parker et al. (2018)					●		1
Catlin et al. (2020)						●	1
Graham-Rowe et al. (2019)						●	1
Grewal et al. (2019)						●	1
Kurth et al. (2020)						●	1
Wang et al. (2017)						●	1
Bardsley et al. (2019)	●	●					2
Bhanot (2017)	●	●					2
Bogard et al. (2020)	●	●					2
Borden et al. (2018)	●	●					2
Charlier et al. (2021)	●	●					2
Crago et al. (2020)	●	●					2
Geiger et al. (2020)	●	●					2
Gillingham & Tsvetanov (2018)	●	●					2
Gossling et al. (2019)	●	●					2
Hayashi et al. (2019)	●	●					2
Henry et al. (2019)	●	●					2
Hodges et al. (2020)	●	●					2
Jessoe et al. (2021)	●	●					2
Myers & Souza (2020)	●	●					2

Article	Education/ Informational feedback	Social norms	Economic incentives	Cognitive biases/ Choice architecture	Goal setting	Emotion	# of Intervention Frameworks
Ornaghi et al. (2018)	•	•					2
Brent & Ward (2019)	•		•				2
Casado et al. (2017)	•		•				2
del Mar Solà et al. (2021)	•		•				2
Dolnicar et al. (2020)	•		•				2
Dur & Vollaard (2019)	•		•				2
Lang & Lanz (2021)	•		•				2
Lang et al. (2021)	•		•				2
Matsukawa (2018)	•		•				2
Rodemeier et al. (2017)	•		•				2
Schleich et al. (2017)	•		•				2
Stojanovski et al. (2020)	•		•				2
van der Werf et al. (2021)	•		•				2
Xu et al. (2018)	•		•				2
Hafner et al. (2019)	•			•			2
Joo et al. (2018)	•				•		2
Pelt et al. (2020)	•				•		2
Vivek et al. (2021)	•				•		2
Tiefenbeck et al. (2018)	•					•	2
Rosenkranz et al. (2017)		•		•			2
Sintov et al. (2019)		•		•			2
Wu & Paluck (2021)		•		•			2

Article	Education/ Informational feedback	Social norms	Economic incentives	Cognitive biases/ Choice architecture	Goal setting	Emotion	# of Intervention Frameworks
Albalate & Gragera (2020)			●	●			2
Ellison et al. (2017)			●	●			2
Castel et al. (2019)			●		●		2
Jensen et al. (2020)			●			●	2
Aydin et al. (2018)	●	●	●				3
Chen et al. (2017)	●	●	●				3
Hafner et al. (2019)	●	●	●				3
Jaeger & Schultz (2017)	●	●	●				3
Pellerano et al. (2017)	●	●	●				3
Sudarshan (2017)	●	●	●				3
Soma et al. (2020)	●		●		●		3
Brent et al. (2020)		●	●		●		3

Appendix 6. Behaviors

The dependent variables for most articles usually measure actual behavior rather than relying on self-reported behavior or behavioral intentions. This is illustrated in Table A1, with 72% of dependent variables representing measured behavior. Articles coded as measured behavior present behavior that is measured directly by the researchers or by a source other than the participants themselves (e.g., a utility company or housekeeping staff). Frequent examples of measured behavior include kWh of electricity used or gallons of water used, which are measured by utility companies that provide the information to researchers. Articles coded as self-reported behavior obtain data about the frequency or amount of a particular behavior directly from participants in the study. Examples of self-reported behavior include Likert scales that ask people to rate the frequency of a particular behavior (Chwialkowska & Flicinska-Turkiewicz, 2021), questions that ask people to report their "family's current water consumption level in a day (in liters or in number of buckets)" (Sengupta, 2020), or estimates of the percentage of a household's vegetables thrown away in the previous seven days (Graham-Rowe et al., 2019). Articles coded as behavioral intention collect data through the use of hypothetical questions or simulations. Examples of behavioral intentions include studies that ask participants how likely they would be to "consider installing an energy efficient heat pump" (Hafner et al., 2019) or how much of a rent increase would people be willing to pay for more energy efficient heating systems (Lang & Lanz, 2021).

Table A1. Type of Behavior

Type of Behavior	Number of Articles	% of Articles
Measured behavior	71	72%
Self-reported behavior	16	16%
Behavioral intention	12	12%
Total	99	100%

Appendix 7. Length of Intervention

The length of intervention indicates how long the intervention itself lasted. It should not be confused with how long data collection lasted, which may be important for determining if an intervention becomes less effective over time. The length of the intervention is likely to be a useful piece of information because it indicates how long will be required to potentially achieve an effect similar to one observed in the published literature. A considerable number of the interventions were completed in one day or less (37%), as can be seen in Table A2. Examples of interventions coded as *day of less* include studies where one letter or postcard was mailed to participants (Lede et al., 2019; Gillingham & Tsvetanov, 2018), one-shot online studies completed with MTurk participants (Hafner et al., 2019), and studies conducted in businesses where customers could be expected to complete their transactions in one day or less (Mookerjee et al., 2021). Interventions coded as *day–week* took longer than a day but a week or less. Examples include interventions that gave a financial incentive for ordering vegetarian lunches each day for a week (Kaiser et al., 2020) and a booklet wherein families staying at a hotel would get stamps for not leaving any food on their plates over the course of their stay that averaged a week or less (Dolnicar et al., 2020). Interventions coded as *week–month* took longer than a week but a month or less. Examples include studies that gave people one month of free public transportation (Friman et al., 2019) and studies that emailed people information about their energy consumption every Friday for a month (Wong-Parodi et al., 2019). Interventions coded as *month–year* took longer than a month but a year or less. Examples include continuously updated in-home electricity usage display monitors installed for eight months (Aydin et al., 2018) and weekly emails about electricity consumption over the course of a university semester (Myers & Souza, 2020). Interventions coded as *longer than a year* took longer than a year. Examples include studies examining the effects of a new (and ostensibly permanent) parking regulation (Albalate & Gragera, 2020) and a program (spanning multiple years) that offered rebate checks for purchasing energy efficient appliances (Schaufele, 2021). One article (Brent et al., 2020) was counted in two categories because they varied the number and timing of letters sent in different intervention conditions.

Table A2. Length of Intervention

**Length of Intervention**	**Number of Articles**	**% of Articles**
Day or less	37	37%
Day–Week	4	4%
Week–Month	14	14%
Month–Year	37	37%
Longer than a year	7	7%
Unspecified	1	1%
Total	99	100%

Appendix 8 Location of the study

As shown in Table A3, most of the studies are field studies conducted in the *real-world (85%)*, and in most of these field studies participants were *unaware* (61%) that they were being observed as part of a research study. This type of research design has the benefit of reducing the Hawthorne effect, thereby increasing the likelihood that interventions will have the same effect if implemented in a non-research setting. If people are unaware that they are in a research study, however, they most likely were unable to provide informed consent, which can pose ethical concerns. All studies conducted in the real world that mentioned people providing consent for participating were coded as aware rather than unaware. None of the studies that did not mention consent being provided mentioned anything about an institutional review board authorizing a waiver of informed consent. A few of the articles relied entirely on participants from *online* participant pools (such as Amazon Mechanical Turk, MTurk) or participated in an *in-lab* environment

Articles that had multiple studies testing the same question were coded based on the single experiment that was judged to be the most real-world in nature. For example, Kamleitner et al. (2019) had multiple MTurk studies in their paper to support their theoretical account of why telling people about an item's past identity increases the item's value[1]. However, they also included a real-world study that involved setting up a popup store. This article was therefore coded as *real-world (unaware)* because customers of the popup store were unaware that their purchasing decisions were part of a research study. We thought this strategy of coding only the most real-world experiment in a set of multiple studies seemed reasonable because including additional online studies in a paper for the purposes of refining a theory would not harm generalizability in the same way as failing to include a real-world implementation of the study. We would not want to give the mistaken perception that real-world studies are rare by piling on additional online studies that are published alongside a well-designed real-world study.[2]

---

[1] They argue that knowing about an item's history induces narrative thoughts, which makes customers feel special.

[2] Imagine if we had instead coded each study rather than the most real-world study within a paper. In Scenario A, there are two single-study real-world experiments in two published papers. This would give us 100% of the Scenario A articles being real-world. In Scenario B, we have the exact same, two published papers as in Scenario A, but each

Table A3. Geographical Location of Study

Location of Study	Number of Articles	% of Articles
Real-world (unaware)	51	52%
Real-world (aware)	33	33%
Online	12	12%
In-lab	6	6%
Total	99	100%

---

article also has three online MTurk studies to better flesh out the theoretical mechanism. If anything, Scenario B is superior to Scenario A because it has all of the same real-world research as Scenario A, but it also has a better grasp on the theory. However, if we counted all studies rather than articles, Scenario B would be coded as only 25% real-world. Counting all studies rather than the most real-world experiment within a single paper would therefore provide a misleading account of the percentage of research that is real-world relative to online or in-lab.

Appendix 9. Control group

Most of the studies (73%) had a between-subjects control group that allowed any effect of the intervention to be compared with the current (no intervention) state of the world. Studies coded as *no intervention* may have also compared multiple interventions and may have also had baseline measurements from prior to the intervention. In other words, studies were coded as *other intervention* or *baseline* only if they did not have a between-subjects control group. Similarly, studies were coded as *baseline* only if they lacked both a *no intervention* and an *other intervention* control group. See Table A4.

An important consideration that is often overlooked is that different types of control groups may be better suited for different research goals. One goal of the present research is to evaluate interventions that could be used to change actual behavior. While a good theoretical understanding of the issue is likely to motivate and inspire the most effective interventions, the effect of exactly how an intervention is implemented may be even more important for applied purposes.

These differences between the types of experiments that are best suited for applied purposes or theoretical understanding are considered frequently in the context of lab-based versus field research. Lab-based research is generally considered to have greater internal validity due to greater experimental control. Researchers in the lab can minimize differences in ambient temperature, distractions, bystanders, etc. more than researchers in the field. This precise control of a variable can allow for stronger inferences about causation than less precisely controlled field studies.

If the effect of the intervention is simply due to differences in expectations (similar to a placebo effect) between the intervention and no intervention control, would people who get the intervention in the future be expected to also have these different expectations? If so, we would still expect that future implementations of the intervention to be effective. This would be true even if the theoretical account is entirely wrong, and the effect is solely due to different placebo-like expectations between the experimental and control groups. In order to better control for placebo effects and different participant expectations, “active” control groups are sometimes proposed as a superior alternative to “no-contact” control groups. If the goal, however, is to

determine the effect of an intervention compared to the current state of the world, it does not make sense to control for expectations that would continue to exist in future implementations of the intervention.

This consideration (about whether the same placebo-like differences in expectations might exist in both the previously published implementation and in future implementations) ties back to our coding about awareness of participation in a research study. As we noted earlier, in most studies participants seemed to be unaware that their behavior was being observed for a research study. We would therefore expect future real-world implementations that are not for research purposes (in which case people would certainly not be aware they are being observed in a research study) to behave similarly to how they behaved in the published research. Hence for applied purposes, the ideal control group for a study that observes the behavior of people who are unaware they are being observed is likely to be a no-intervention control.

There, however, may be ethical considerations about collecting data from people who have not consented to participate in research. Institutional review boards therefore need to provide waivers of informed consent in order to ensure that the best interests of human participants are being considered. Situations in which only participants in the experimental condition provide informed consent (and are therefore aware of participation) begin to create situations wherein differing participant expectations must be considered. If only participants in the experimental condition know they are being observed and this awareness causes them to behave more environmentally friendly, future real-world implementations that are not for research purposes might fail due to this mismatch between the research and implementation.

The previous example illustrates problems that can arise due to a lack of control. Namely, differences in awareness of being in a research study between conditions. Let's now consider problems that can arise due to too much experimental control. Doorhangers (e.g., Jaeger & Schultz, 2017; Bhanot, 2021) and mailers (Brent et al., 2020; Hodges et al., 2020) are common interventions used to reduce waste. Should the research attempt to control for the effect of getting a doorhanger or a mailer? If the goal of the research is to answer a theoretical question about the effect of specific information presented on the doorhanger, then it would be quite reasonable to want to control for the effect of getting a doorhanger itself. However, if the goal of

the research is to predict the effect of the intervention compared to the current state of the world, we would say no. The doorhanger is part of the intervention package that would be used in a future implementation, so controlling out the effect of the doorhanger would provide a misleading picture of the expected effect on an intervention compared to the current state of the world.

What if getting a doorhanger makes people *think* that they are being observed, which then causes them to behave differently, wouldn't it then make sense to control for the doorhanger? If the doorhanger itself makes people think they are being observed, we would have every reason to believe that a future implementation of this doorhanger would similarly cause people to think they are being observed and therefore behave in line with this thinking they are being observed. We therefore would still not want to control out the effect of the door hanger if the goal is to predict how the intervention will change behavior compared to the current state of the world.

Table A4. Type of Control Group

Control Group	Number of Articles	% of Articles
No intervention	72	73%
Other intervention	19	19%
Baseline	8	8%
Total	99	100%

Appendix 10. Study Randomization Design

Most experiments (77%) had random assignment to condition and are therefore considered to be "true" experiments rather than quasi-experiments (See Table A5). Random assignment is important for determining causation because with large enough sample sizes, pre-existing differences between groups should be equated on average. The goal of the present research is to identify interventions that have proven to be effective in previous work so that these interventions can be used to change behavior. For this reason, it is necessary to establish a causal link between the intervention and the behavior.

A number of research designs can fall under the umbrella of quasi-experiment, and they are likely to vary in how difficult it is to determine causation from the findings. Determining causation is the most difficult in cases wherein participants sort themselves into experimental or control groups. In one study (Wang et al, 2021), all participants who opted into a demand response energy program were placed into the experimental group whereas all remaining participants were placed into the control group. Because of this self-selection into conditions, no matter how large the sample size, participants would not be expected to be equated on pre-existing differences.

Other studies coded as quasi-experiment include natural experiments such as the effect of a grocery store banning single-use plastic bags (Isbanner et al. 2021) or a city implementing a new parking regulation (Albalate & Gragera, 2020). New laws and policies are likely to come into existence because the community supports these types of policies, which means that the members of communities that support such policies may not be the same as comparison communities that do not support those policies. Moreover, laws and policies often go into effect when support is on an increasing (rather than decreasing) trajectory. This means that a behavior might still be expected to change over time in a manner consistent with this trajectory even if the policy had not gone into effect. Despite these limitations, natural experiments can still provide valuable insights into behavior.

Table A5. Study Randomization Design

Random Assignment	Number of Articles	% of Articles
Experiment	76	77%
Quasi-experiment	23	23%
Total	99	100%

Appendix 11. Sample size (N)

The average sample size was $N = 4299$. This value, however, is considerably skewed right with a median sample size of $N = 336$. Figure A1 Panel A shows the distribution of N values for all articles. Because of the considerable right skew of the data, we also plotted the distribution of N values for the 72 articles with sample sizes less than 1,000 in Figure A1 Panel B.

Figure A1.

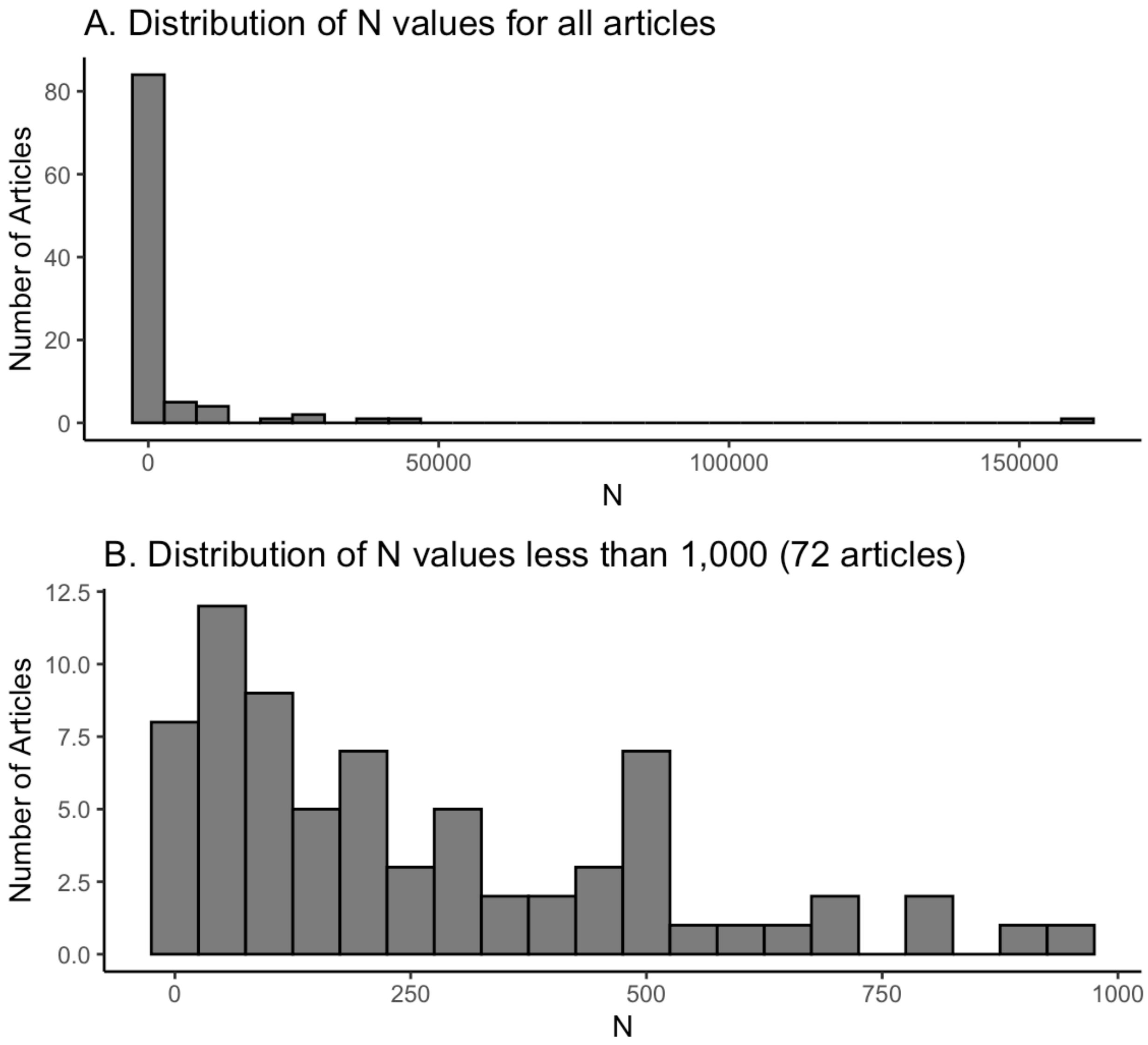

Appendix 12. Country of Participants

The most common country for participants was the United States (27%) followed by Germany (9%) and United Kingdom (9%) as can be seen in Table A6. Although the distribution of countries appears to be somewhat dispersed, many countries are underrepresented or overrepresented relative to their population. For example, only 3% of articles have participants from China, while China has 18% of the world population (World Bank, 2021). Alternatively, 27% of study participants are from the United States while the United States has 4% of the world population (World Bank, 2021). One article (Clayton et al., 2021) included participants from both the United States and Canada and was therefore counted for both countries.

Table A6. Country of Participants

Country	Number of Articles	% of Articles
United States	27	27%
Germany	9	9%
United Kingdom	9	9%
Canada	6	6%
Australia	4	4%
Netherlands	4	4%
Sweden	4	4%
China	3	3%
France	3	3%
India	4	4%
Spain	4	4%
Switzerland	3	3%
Chile	2	2%

Japan	2	2%
Poland	2	2%
Unspecified European Country	2	2%
Other	12	12%
Total	99	100%

SUPPLEMENTARY MATERIAL REFERENCES

Albalate, D., & Gragera, A. (2020). The impact of curbside parking regulations on car ownership. *Regional Science and Urban Economics*, *81*, 103518.

Aydin, E., Brounen, D., & Kok, N. (2018). Information provision and energy consumption: Evidence from a field experiment. *Energy Economics*, *71*, 403-410.

Bhanot, S. P. (2021). Isolating the effect of injunctive norms on conservation behavior: New evidence from a field experiment in California. *Organizational Behavior and Human Decision Processes*, *163*, 30-42.

Brent, D. A., Lott, C., Taylor, M., Cook, J., Rollins, K., & Stoddard, S. (2020). What Causes Heterogeneous Responses to Social Comparison Messages for Water Conservation? *Environmental and Resource Economics*, *77*(3), 503-537.

Chwialkowska, A., & Flicinska-Turkiewicz, J. (2021). Overcoming perceived sacrifice as a barrier to the adoption of green non-purchase behaviours. *International Journal of Consumer Studies*, *45*(2), 205-220.

Clayton, J., Devine, A., & Holtermans, R. (2021). Beyond building certification: The impact of environmental interventions on commercial real estate operations. *Energy Economics*, *93*, 105039.

Dolnicar, S., Juvan, E., & Grün, B. (2020). Reducing the plate waste of families at hotel buffets–A quasi-experimental field study. *Tourism Management*, *80*, 104103.

Friman, M., Maier, R., & Olsson, L. E. (2019). Applying a motivational stage-based approach in order to study a temporary free public transport intervention. *Transport Policy*, *81*, 173-183.

Gillingham, K., & Tsvetanov, T. (2018). Nudging energy efficiency audits: Evidence from a field experiment. *Journal of Environmental Economics and Management*, *90*, 303-316.

Graham-Rowe, E., Jessop, D. C., & Sparks, P. (2019). Self-affirmation theory and pro-environmental behaviour: Promoting a reduction in household food waste. *Journal of Environmental Psychology*, *62*, 124-132.

Hafner, R., Elmes, D., & Read, D. (2019). Exploring the role of messenger effects and feedback frames in promoting uptake of energy-efficient technologies. *Current Psychology*, *38*(6), 1601-1612.

Hafner, R., Elmes, D., Read, D., & White, M. P. (2019). Exploring the role of normative, financial and environmental information in promoting uptake of energy efficient technologies. *Journal of Environmental Psychology*, *63*, 26-35.

Hodges, H., Kuehl, C., Anderson, S. E., Ehret, P. J., & Brick, C. (2020). How managers can reduce household water use through communication: A field experiment. *Journal of Policy Analysis and Management*, *39*, 1076-1099.

Isbanner, S., Algie, J., & Reynolds, N. (2021). Spillover in the context of forced behaviour change: observations from a naturalistic time-series study. *Journal of Marketing Management*, *37*(7-8), 703-731.

Jaeger, C. M., & Schultz, P. W. (2017). Coupling social norms and commitments: Testing the underdetected nature of social influence. *Journal of Environmental Psychology*, *51*, 199-208.

Kaiser, F. G., Henn, L., & Marschke, B. (2020). Financial rewards for long-term environmental protection. *Journal of Environmental Psychology*, *68*, 101411.

Kamleitner, B., Thürridl, C., & Martin, B. A. (2019). A Cinderella story: How past identity salience boosts demand for repurposed products. *Journal of Marketing*, *83*(6), 76-92.

Lang, G., & Lanz, B. (2021). Energy efficiency, information, and the acceptability of rent increases: A survey experiment with tenants. *Energy Economics*, *95*, 105007.

Lede, E., Meleady, R., & Seger, C. R. (2019). Optimizing the influence of social norms interventions: Applying social identity insights to motivate residential water conservation. *Journal of Environmental Psychology*, *62*, 105-114.

Mookerjee, S., Cornil, Y., & Hoegg, J. (2021). From Waste to Taste: How "Ugly" Labels Can Increase Purchase of Unattractive Produce. *Journal of Marketing*, *85*(3), 62-77.

Myers, E., & Souza, M. (2020). Social comparison nudges without monetary incentives: Evidence from home energy reports. *Journal of Environmental Economics and Management*, *101*, 102315.

Schaufele, B. (2021). Lessons from a utility-sponsored revenue neutral electricity conservation program. *Energy Policy*, *150*, 112157.

Wang, Z., Zhao, W., Deng, N., Zhang, B., & Wang, B. (2021). Mixed data-driven decision-making in demand response management: An empirical evidence from dynamic time-warping based nonparametric-matching DID. *Omega*, *100*, 102233.

Wong-Parodi, G., Krishnamurti, T., Gluck, J., & Agarwal, Y. (2019). Encouraging energy conservation at work: A field study testing social norm feedback and awareness of monitoring. *Energy Policy*, *130*, 197-205.

World Bank. (2021). Retrieved from https://databank.worldbank.org/data/download/POP.pdf
